# Addressing the Reproducibility Crisis in Science: It’s About Time

**DOI:** 10.1016/j.jacbts.2024.09.005

**Published:** 2024-10-28

**Authors:** Martin Young, Douglas L. Mann

**Figure:**
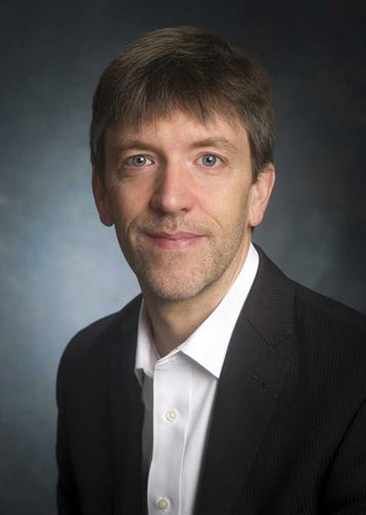

**Figure:**
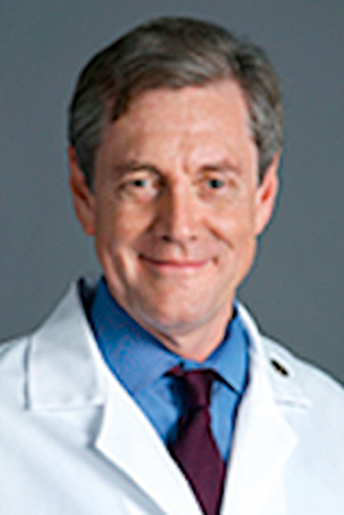

In his book "Rigor Mortis: How Sloppy Science Creates Worthless Cures, Crushes Hope, and Wastes Billions," James Harris^1^ discusses issues such as poor experimental design, improper methods, and inadequate statistical methodology as major contributors to the reproducibility crisis in science. However, recent studies have shown that biological variability also plays a significant role in the reproducibility of both preclinical and clinical research. This includes chronobiology. The term circadian rhythms is used to describe 24-hour cycles in physiological, behavioral, and molecular processes that are driven by internal timekeeping mechanisms known as circadian clocks. In this Editor’s Page we will discuss how circadian biology can contribute to biological variability and discuss why it should be considered when designing experiments in both animal models and human studies.

## Chronobiology

In mammals, circadian governance of biological processes is mediated by a collection of local clocks within virtually all cells in the body, including neurons, hepatocytes, cardiomyocytes, and immune cells. Although these peripheral clocks operate independently, they are entrained by environmental cues such as light, feeding, and physical activity, thus ensuring internal synchronization between organs, as well as synchronization with the environment. At the molecular level, circadian clocks consist of a set of core clock components, including CLOCK, BMAL1, Period (PER1/2/3), and Cryptochrome (CRY1/2), that form transcriptional-translational feedback loops to generate temporal control over cellular processes.^2^

Fundamental physiological processes, including metabolism, immune responses, cardiovascular function, hormone secretion, and sleep-wake cycles, are all circadian regulated. As such, circadian biology is not merely a temporal backdrop but an active participant in determining the efficacy of biological systems at different times of the day. Failure to account for these rhythmic processes can lead to significant biological variability, particularly in studies that involve metabolism, pharmacology, immunology, and disease models.

The circadian clock also regulates cardiovascular function, including heart rate, blood pressure, and vascular tone. Studies have shown that the risk of cardiovascular events, such as heart attacks and strokes, peaks in the early morning, coinciding with a surge in blood pressure and platelet aggregation. Moreover, it has been estimated that 50% of the world’s top-selling drugs target clock-controlled proteins, and, of these drugs, 50% have half-lives that are <24 hours. These “moving targets” impact the time of day dependence of drug effectiveness, termed chronopharmacology. Similarly, various cardiovascular-relevant biomarkers have established 24-hour rhythmicity. Experiments that aim to model cardiovascular diseases or test cardiovascular drugs should account for these circadian variations.

## Chronobiology as a Source of Biological Variability

The circadian clock introduces temporal variability that can significantly affect experimental outcomes. This variability can be divided into 2 main categories: intraindividual and interindividual variability. Intraindividual variability refers to the fluctuations in biological processes that occur within a single organism over the course of the day. For example, an individual’s metabolic rate, immune responsiveness, or drug metabolism varies depending on the time of day. If experiments are conducted at different times without accounting for circadian rhythms, the results may appear inconsistent or irreproducible. Interindividual variability arises from differences in the circadian phase between individuals. This can be influenced by genetic differences (such as chronotype), lifestyle factors (such as sleep and eating patterns), and environmental cues (such as light exposure). Some individuals may be "morning types" (early chronotypes), whereas others are "evening types" (late chronotypes), leading to significant differences in the timing of circadian-regulated processes.

Ignoring circadian rhythms can, therefore, result in misleading conclusions. For example, a drug that appears ineffective when administered in the morning may be highly effective when given in the evening due to circadian regulation of its metabolism and/or rhythms in the drug target. Similarly, experiments performed at distinct times of day by different investigative teams may yield conflicting results, not because of inherent flaws in the study design but because of unrecognized circadian variability.

## Incorporating Circadian Considerations into Experimental Design

Given the pervasive influence of circadian rhythms on biological systems, it is essential that researchers incorporate circadian considerations into experimental design to reduce variability and improve reproducibility. Here are some key strategies to achieve this:

1) Standardize the Timing of Experiments: One of the simplest ways to reduce circadian-related variability is to conduct all experiments at the same time of day. This ensures that all animals or human subjects are in the same circadian phase when the measurements are taken, reducing intraindividual variability.
2) Record and Report Time of Day: In addition to standardizing the timing of experiments, researchers should also record and report the time of day when experiments are conducted. This allows for better comparison across studies and provides crucial context for interpreting the results.
3) Use Time-Dependent Analyses: For experiments in which circadian rhythms are likely to play a role, researchers should consider performing time-course studies. By taking measurements at multiple time points throughout the day, researchers can capture the dynamic fluctuations in biological processes and identify circadian influences on their outcomes. Consider ambulatory blood pressure monitoring as an example, to expose nondipping hypertension.
4) Use Animal Models With Known Circadian Phenotypes: In animal studies, it is important to use models with well-characterized circadian rhythms. For example, mice are nocturnal, and their activity patterns differ from those of diurnal humans. Understanding the circadian biology of the chosen model organism is crucial for designing experiments that can be accurately translated into human physiology. Consider interventions during the dark period in rodent-based studies as an example, when investigating the impact of drugs, diets, or exercise on the cardiovascular system.

## Conclusions

Chronobiology is a major contributor to biological variability, influencing a wide range of physiological processes across different times of the day. Ignoring circadian rhythms in experimental design can lead to misleading conclusions, reduced reproducibility, and failed translation from preclinical to clinical studies. By incorporating circadian considerations, such as standardizing experimental timing, conducting time-course analyses, and accounting for individual differences in chronotype, researchers can reduce biological variability and improve the reproducibility of their findings. Recognizing the circadian nature of biological systems is essential for advancing our understanding of complex physiological processes and developing more effective cardiovascular therapeutic interventions.
